# Effect of Sand Addition on Laterite Soil Stabilization

**DOI:** 10.3390/ma17246033

**Published:** 2024-12-10

**Authors:** Bárbara Drumond Almeida, Lisley Madeira Coelho, Antônio Carlos Rodrigues Guimarães, Sergio Neves Monteiro

**Affiliations:** 1 Department of Fortification and Construction, Military Institute of Engineering-IME, Praça General Tibúrcio, 80, Urca, Rio de Janeiro 22290-270, Brazil; madeiralisley@gmail.com (L.M.C.); guimaraes@ime.eb.br (A.C.R.G.); 2 Department of Materials Science, Military Institute of Engineering-IME, Praça General Tibúrcio, 80, Urca, Rio de Janeiro 22290-270, Brazil; snevesmonteiro@gmail.com

**Keywords:** soil stabilization, laterite, sand, mechanical behavior

## Abstract

Lateritic soils, particularly abundant in tropical regions, have been successfully used in the construction of unbound layers of flexible pavements in Brazil since the 1970s. Despite their potential, these soils are often discarded or only recommended after stabilization processes, based on traditional parameters such as gradation requirements and Atterberg limits. This study investigates the mechanical characteristics of a lateritic soil from Roraima, focusing on its resilient modulus and permanent deformation properties, assessed through repeated load triaxial tests. Specifically, this research examines the effect of adding 20% sand on the mechanical behavior of the material. The results indicate that sand addition did not significantly improve the mechanical performance. The laterite–sand mixture exhibited an average resilient modulus (RM) of 744 MPa, lower than the 790 MPa of pure lateritic soil, suggesting that pure laterite remains suitable for pavement applications. Furthermore, the permanent deformation analysis revealed that the mixture with sand experienced nearly twice the plastic strain compared to pure laterite, which demonstrated superior accommodation under repeated loading. In the shakedown analysis, pure laterite exhibited a more stable performance, indicating greater durability in pavement applications. These findings highlight the importance of understanding the mechanical behavior of lateritic soils beyond conventional testing methods, emphasizing the potential of pure laterite as a viable alternative to enhance the strength and durability of pavement structures.

## 1. Introduction

Laterites or lateritic gravels are low-cost materials widely found in tropical countries and have shown significant potential for use in paving. Laterites are generally highly weathered and altered residual soils with a low silica content that contain a sufficient concentration of iron and aluminum sesquioxides which allows them to be cemented to some degree [1]. In Brazil, these materials are especially abundant in the northern region and have been successfully used in unbound layers of flexible pavements since the 1970s. However, despite their potential, laterites are often discarded or recommended only after stabilization processes based on traditional parameters such as particle size requirements and Atterberg limits. Several studies have highlighted the importance of better understanding the mechanical behavior of laterites beyond typical tests such as California Bearing Ratio (CBR) tests, Atterberg limit assessments, and others required by technical specifications. Advanced testing methods, such as repeated load triaxial (RLT) tests, have gained prominence for evaluating the resilient modulus and simulating in-service stress conditions, providing a more comprehensive assessment of soil performance under traffic loads [2,3,4,5,6,7]. These methods allow for the characterization of nonlinear and stress-dependent behavior, crucial for modern pavement design approaches.

Netterberg [8] pointed out that laterites have not been fully utilized in the upper layers (base and sub-base) of paved roads due to shortcomings in meeting technical standards, such as a high plasticity index (PI), difficulties in achieving gradation within standard curves, low CBR values, and unsatisfactory results in Los Angeles abrasion tests. Nonetheless, studies like those by Cardoso [9] and Grace [10] confirm that laterites, even when failing to meet all technical criteria, have shown satisfactory performance in pavement layers in Brazil and other tropical regions.

The need for standardized natural materials, including lateritic soils, has posed significant challenges for road construction. To address these limitations, soil improvement techniques have been explored, with stabilization through cement and other additives being commonly adopted [11]. However, the potential for using lateritic soils in their natural state remains underexplored. For instance, studies by Guimarães et al. [2] and Galhardo et al. [12] demonstrate the high resilient modulus and low permanent deformation of lateritic soils, even without strict adherence to conventional standards. Similarly, work by Veloso et al. [13] further validates the mechanical suitability of laterites for pavement applications.

Among the alternative techniques for improving the gradation and geotechnical properties of lateritic soils, the addition of sand has been highlighted in various studies. For instance, Consoli et al. [14] demonstrated that the combination of sand addition, portland cement incorporation, and densification by compaction significantly improved the strength and durability of lateritic soils. Similarly, Madu [15] and Madjadoumbaye et al. [16] investigated the use of sand–laterite mixtures in road construction and observed improvements in strength, plasticity index, and CBR. Okonkwo et al. [17] also reported an enhanced stabilization of lateritic soils with portland cement and sand, addressing challenges in road pavement construction due to a shortage of high-quality materials.

This study focuses on a lateritic soil from Roraima, a region rich in these materials. Observations from local construction projects suggest the potential use of adding sand to laterites in order to meet grading and geotechnical requirements. This research aims to evaluate the impact of adding 20% sand on the mechanical properties of this lateritic soil. The resilience modulus and permanent deformation will be assessed through repeated-load triaxial testing. By comparing the results for both pure and sand-modified laterite, this study will determine whether the sand modification significantly improves its properties or if the natural lateritic soil is already suitable for use in pavement base and sub-base layers.

## 2. Materials and Methods

### 2.1. Materials

**Figure 1:**
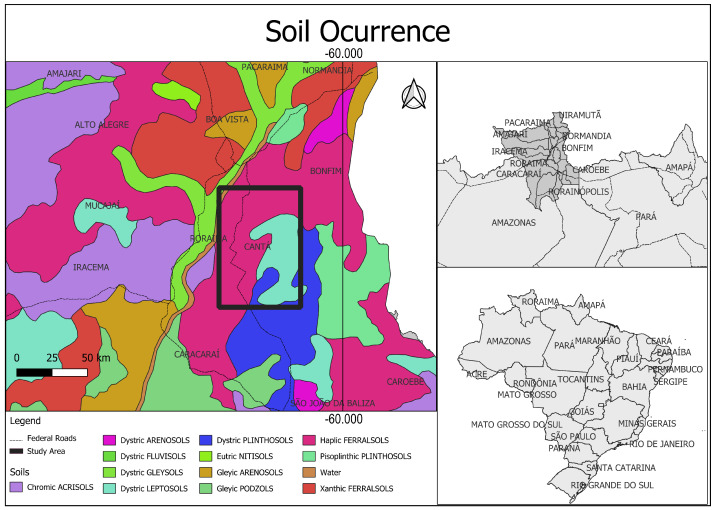
Soil map of Brazil highlighting the study area. Prepared with data from the Brazilian Agricultural Research Corporation—EMBRAPA [19].

Two samples were analyzed to evaluate the influence of sand addition on the behavior of laterite soil: one composed exclusively of laterite soil and another with a 20% addition of sand, referred to throughout this study as laterite soil and laterite soil + sand, respectively. Representative samples of the materials, including sand and laterite, were collected along a road in the Roraima region, as illustrated in Figure 1. The choice of a 20% sand addition was informed by technical observations in the region, aiming to adjust the soil’s granulometry to meet geotechnical standards commonly applied in pavement design. This area is part of the Legal Amazon territory, which plays a crucial role nationally and globally in terms of biodiversity, environmental preservation, and national defense, as highlighted by Mello and Artaxo [18]. The region lies within sedimentary basins and unconsolidated covers of the Boa Vista depression and encompasses basement rocks in the styles of the residual plateaus of Roraima, the Rio Branco–Rio Negro pediplain, and the residual plateaus of northern Amazonia. The predominant climate is equatorial tropical, with average temperatures exceeding 18 °C throughout the year and a semi-humid regime, featuring a dry season lasting 4 to 5 months.

### 2.2. Methods

The samples were dried, separated, and quartered, with a portion allocated for particle size distribution analysis according to the DNER-ME 083 [20], DNER-ME 080 [21], and DNIT 098 [22] standards for material classification. Liquid limit and plastic limit tests (Atterberg limits) were also performed following the DNER-ME 082/94 [23] standard.

For international comparison, the samples were classified according to the SUCS (Soil Use and Classification System) classification, as well as according to the TBR (Transportation Research Board) system from AASHTO (the American Association of State Highway and Transportation Officials). The AASHTO classification enables the assessment of soil suitability for use as a roadbed based on physical and conditional parameters [24].

Compaction tests were performed on both the laterite + sand mixture and the pure laterite using the modified Proctor energy method, using a tripartite mold, to determine the optimum moisture content (Wot) and the maximum dry density ($\rho_{\mathrm{dmax}}$).

#### 2.2.1. MCT (Miniature, Compacted, Tropical) Methodology

The MCT classification (DNIT 259 [25]) is based on indices obtained from tests conducted on small-sized specimens. The critical tests for determining these indices are the mini-MCV compaction test (DNIT 258 [26]) and the mass loss test by water immersion (DNER-ME 256 [27]). The mini-MCV (Moisture Condition Value) compaction method generates a family of compaction curves according to Nogami and Villibor [28] and adapted from Parsons’ procedure. These curves allow for the determination of two key indices: the c’ index, related to the particle size distribution, and the d’ index, associated with the slope of the dry branch of the compaction curve at 12 blows. Using these indices, along with the mass loss by immersion (Pi) test result, allows the soils under study to be classified according to the position of the determined indices on the standard classification abacus.

The MCT classification divides soils into two significant groups: lateritic behavior soils, classified as “L”, and non-lateritic behavior soils, classified as “N”, as described by Nogami and Villibor [28]. The mini-MCV compaction test procedure, prescribed by the road standard DNER-ME 258 [26], involves compacting the soil fraction passing through the 2.0 mm sieve. The compaction process follows the number of blows (n) specified in the standard and is halted when the difference between the height of the specimen compacted with “4n” blows and that compacted with “n” blows is less than 2.0 mm. At the end of the test, families of compaction curves and mini-MCV curves are generated, from which the necessary data for the classification procedure can be extracted. For the mass loss by water immersion test, the DNER-ME 258 standard [26] specifies that this parameter is determined by quantifying the mass lost from the previously compacted specimen after immersion in water for a minimum of 20 h. During immersion, the specimen is placed horizontally, with the top protruding 1.0 cm above the edge of the metallic compaction mold.

Classification tests were performed for each studied soil, focusing solely on the fine fraction (passing through the 2 mm sieve) according to the MCT methodology. It is important to note that the MCT classification of the soils in this study, identified as granular soils, is presented for illustrative purposes only. The classification of granular soils, such as laterite and the laterite–sand mixture, is not considered valid due to their specific characteristics. However, the classification of the sand is applicable. The fine-grained particles contain iron and aluminum oxides and undergo cementation, indicating potential effects on the material’s behavior.

#### 2.2.2. Scanning Electron Microscopy (SEM), Energy-Dispersive X-Ray Spectroscopy (EDX), and X-Ray Diffraction (XRD)

The samples were morphologically analyzed with SEM using the QUANTA FEG 250 microscope manufactured by FEI (Brno, Czech Republic). The samples, including laterite soil and laterite soil + sand, were coated with gold utilizing a Leica ACE600 high-vacuum coating chamber. Both belonging to the electron microscopy laboratory (LME) of the military institute of engineering—(IME), Rio de Janeiro, Brazil. SEM analysis was carried out under the following parameters: electron beam power of 20 kV, working distance ranging between 10.5 and 13 mm, spot size of 5, and image magnification at 100× and 1000×, utilizing the secondary electron detector. To perform the XRD analysis, the samples of ateritic soil, lateritic soil with sand, and sand were inserted into a monocrystalline silicon substrate. The analysis was performed using the Xpert Pro MRD System equipment from PANalytics (Vancouver, BC, Canada) with cobalt Kα radiation (1.789 A), at a scan speed of 4°/min and a power of 40 mA × 40 kV and scanning from 20° to 55°. The tests were conducted in line focus configuration, using the X’Pert Data Collector software, version 2.2j (2010), to input the equipment’s operating parameters. The diffraction data was processed using the X’Pert HighScore Plus software, version 2.0a (2004).

#### 2.2.3. Mechanical Analysis

The mechanical analysis involved tests to determine the fundamental properties of the soil, including the resilience modulus and permanent deformation. RLT tests were conducted to simulate the behavior of the material under traffic conditions.

Resilient Modulus

**Figure 2:**
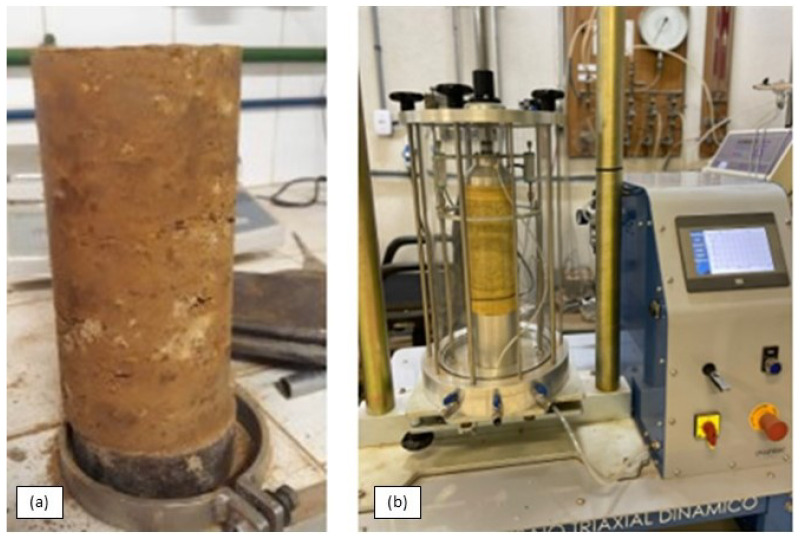
Repeated load triaxial test test. (**a**) Molded laterite specimen; (**b**) dynamic triaxial equipment.

The resilient modulus test was conducted according to the standard test method of the DNIT 134 Brazilian standard [29], using dynamic triaxial testing equipment (Owntec-MS-151, Santa Cruz do Sul - RS, Brazil). The sample compaction was performed in a tripartite cylindrical mold with a diameter of 100 mm and a height of 200 mm. Ten layers of 2 cm each were compacted, with 21 blows each, using modified compaction energy according to the DNIT 443 Brazilian standard [30]. During the RM test, eighteen pairs of confining stresses ($\sigma_{3}$) and deviant stress ($\sigma_{d}$) were applied after the conditioning phase of the specimen. The load cycle lasted 1 s, with 0.1 s of load application and a frequency of 1 Hz. In the conditioning stage, the specimens were exposed to three sets of stresses and subjected to 500 load cycles for each set. Subsequently, they were subjected to 18 additional stresses, with 100 load cycles for each set, totaling 3300 cycles per test. Figure 2 shows the molded latelite specimen outside and inside the dynamic triaxial equipment.

Permanent Deformation (PD)

In the permanent deformation test, repeated load cycles were applied to evaluate each specimen’s behavior under different stress states. The molding of the specimens for the PD test followed the same procedure as the resilient modulus test and complied with the Brazilian standard DNIT 443 [30], and was also carried out on the dynamic triaxial equipment. For this study, the stress pairs shown in Table 1 were used, based on the British standard BS EN 13286-7 [31], with some adjustments made due to the load limitations of the equipment used for the triaxial tests.

In addition, the results were evaluated according to the shakedown theory [32,33,34]. The shakedown concept has been used to describe the behavior of many structures in engineering under cyclic or repeated loading. According to the shakedown theory, there is a critical level of stress that separates stable conditions from unstable conditions. For the evaluation of accommodation, behavior levels are classified as type A, B, C, or AB, with the AB classification being an improvement introduced by Dawson and Wellner [32] and Werkmeister [33], and also discussed by Motta and Guimarães [34], for the study of road pavement:

(A) Shakedown or Plastic Accommodation: In this behavior level, the material exhibits plastic deformations during a finite number of applications of the stress pair and, after the post-compaction period, responds entirely elastically. From this transition, where the material responds only elastically to the applied stresses, it is said that it has entered shakedown.

(B) Intermediate Response Level: The material’s behavior at this level shows high deformation during the initial load cycles and, over subsequent applications, tends to reduce its deformation and assume a more uniform rate. The number of cycles needed for the material to assume a constant deformation rate depends on the material’s mechanical characteristics and the load application amplitude.

(C) Collapse: This is the level at which the response to load application is always plastic. The material deforms considerably with each applied load cycle, with no tendency for stabilization.

(AB): At this level, the material shows significant initial deformations followed by plastic accommodation. This was detected by Guimarães and Motta [34] for fine Brazilian soils.

**Figure 3:**
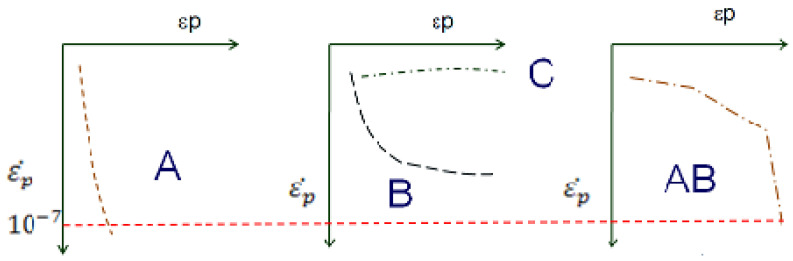
Diagram identifying the three levels of behavior in shakedown research adapted from DNIT 179) [35]. * Indicates tests not carried out, according to the limitations of the scope of the study.

An analysis of these behavior models can be observed in Figure 3.

## 3. Results and Discussion

### 3.1. Characterization of Materials

**Figure 4:**
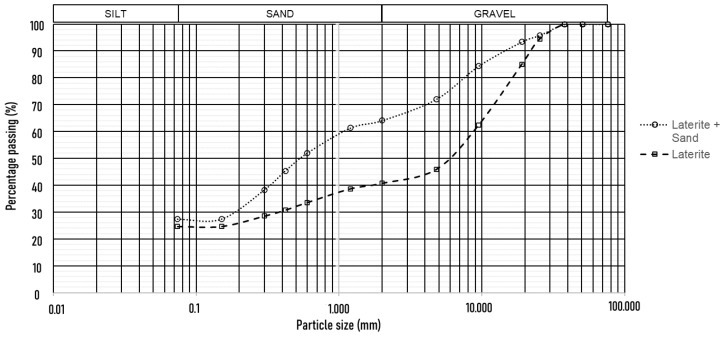
Material grain size distribution.

The grain size distribution, illustrated in Figure 4, and the geotechnical characteristics presented in Table 2 highlight the variation between the analyzed samples. According to the TBR system, the pure laterite was classified as A-2-7, indicating intermediate properties with high plasticity, exhibiting a plasticity index (PI) of 23%. In contrast, the laterite–sand mixture was classified as A-2-4, reflecting an improved granulometric composition and lower plasticity (PI of 10%). Based on Jenkins’s classification, as cited by Caputo [36], soils are categorized as low-plastic (1 < PI < 7), medium-plastic (7 < PI < 15), or highly plastic (PI > 15). Thus, pure laterite is considered highly plastic, whereas adding sand reduces its plasticity to a medium level, enhancing its stability and suitability for pavement applications.

According to the USCS categorization, pure laterite was classified as GC (clayey gravel), while the laterite–sand mixture was classified as SC (clayey sand). This distinction suggests that adding sand improves the particle size distribution and reduces plasticity, potentially resulting in a more stable and efficient material for pavement layers, considering traditional classifications. However, further studies on the mechanical behavior of these soils are essential to validate their use and ensure pavement durability.

The table below also presents the maximum dry density ($\rho_{\mathrm{dmax}}$) and optimum moisture content (Wot) for both materials. The pure laterite exhibited a $\rho_{\mathrm{dmax}}$ of 1.99 g/${\mathrm{cm}}^{3}$ and its Wot value was 13.1%, indicating its suitability for compaction at relatively high moisture levels. Meanwhile, the laterite–sand mixture demonstrated a higher $\rho_{\mathrm{dmax}}$ of 2.16 g/${\mathrm{cm}}^{3}$ and a lower Wot of 9.86%, emphasizing the change in compaction characteristics due to the addition of sand.

### 3.2. MCT Methodology

**Figure 5:**
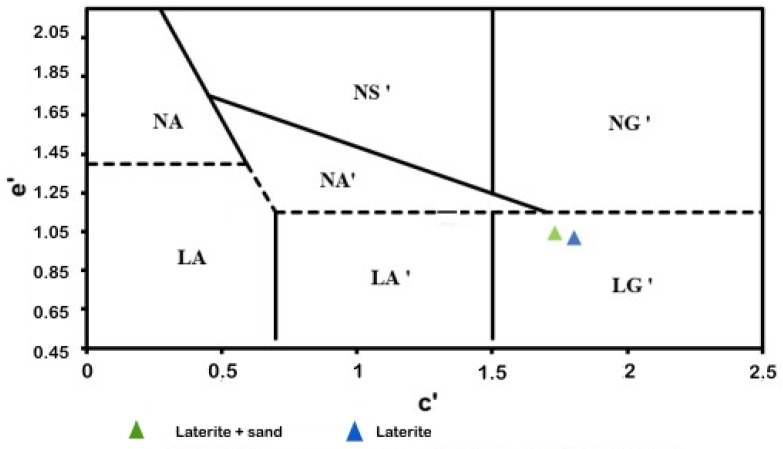
MCT classification.

The classification results are presented in Figure 5. The fine fraction of the soils, including the pure laterite and the laterite–sand mixture, was classified as LG’. While this classification provides an overview of the expected behavior of the materials, it should be interpreted with caution, as the primary focus of this research was to understand the mechanical behavior of the materials rather than their formal classification. Even with the addition of sand, the material remained classified as LG’ because the test was conducted exclusively on the fine fraction (passing through the 2 mm sieve) following the MCT methodology.

### 3.3. SEM, EDX, and XRD of Laterite and Laterite + Sand

**Figure 6:**
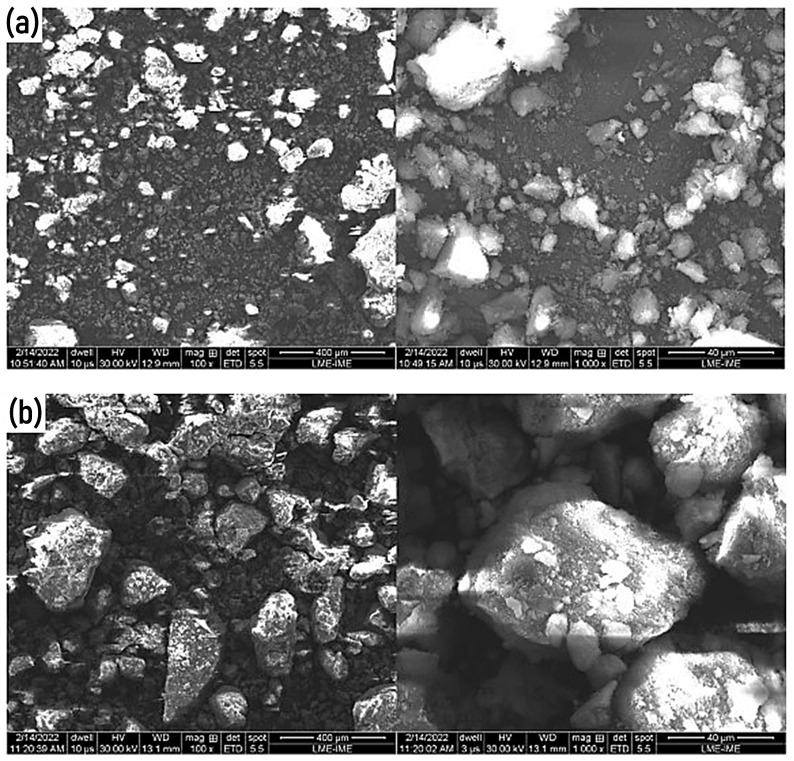
Scanning electron microscopy (SEM) images of samples at different magnifications: (**a**) pure laterite magnified by 100×, showing the overall particle distribution, and 1000×, highlighting the detailed morphology of the particles; (**b**) laterite + sand magnified by 100×, showing the overall particle distribution, and 1000×, highlighting the detailed morphology of the particles.

As proposed by Villibor et al. [37], fine lateritic soils exhibit grains that are poorly individualized as they are bound by an amorphous mass. The grain boundaries are distinctly rounded, with indications of internal voids, giving them a texture similar to “popcorn” or “sponge”. These characteristics are confirmed by the results shown in Figure 6, which illustrates the SEM images of the pure laterite and laterite + sand sample fractions with magnification degrees of 100 and 1000×.

The results of the SEM tests for the fine fraction of pure laterite are presented in Figure 6a, where the “popcorn” or “sponge” texture mentioned by Villibor et al. [37] can be observed. These obtained images are also consistent with the findings of Veloso et al. [13] and Subaer et al. [38].

Figure 6b shows the laterite–sand mixture, where more angular particles can be identified, characteristic of fine soils according to Veloso et al. [13]. These angular particles resemble the sand grains added to the gravelly lateritic soil.

**Figure 7:**
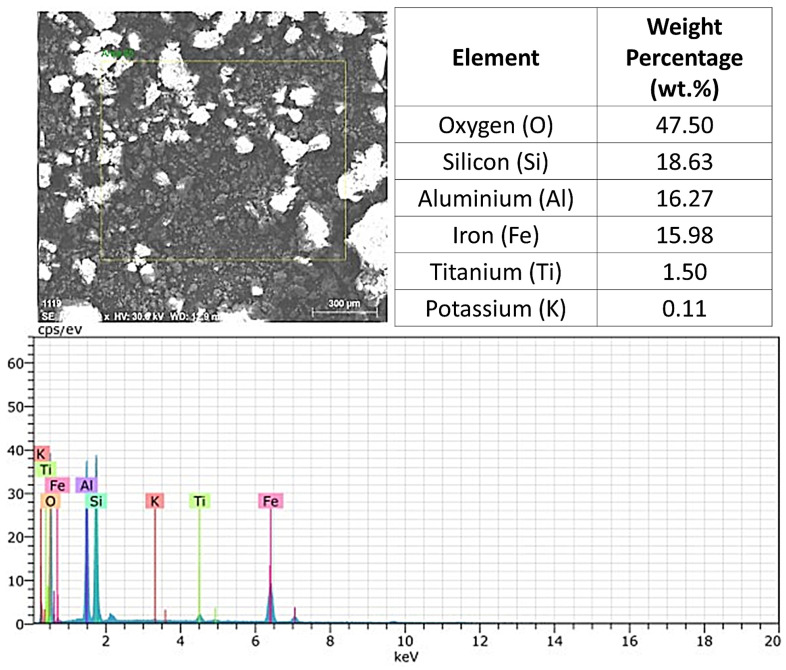
EDX analysis of pure laterite.

The EDX analysis of the pure laterite revealed the presence of the following elements and their respective percentages: O (47.50%), Si (18.63%), Al (16.27%), Fe (15.98%), Ti (1.50%), and K (0.11%), as illustrated in Figure 7. The predominant presence of oxygen, silicon, aluminum, and iron is consistent with other findings reported in the literature, which classifies lateritic soils as rich in aluminum and iron oxides, as indicated by Sivarajasingham et al. [39] and corroborated by Blight [1]. The high oxygen concentration is typical of materials containing water in their structure, while the significant presence of silicon reflects the low silica content characteristic of lateritic soils [1].

**Figure 8:**
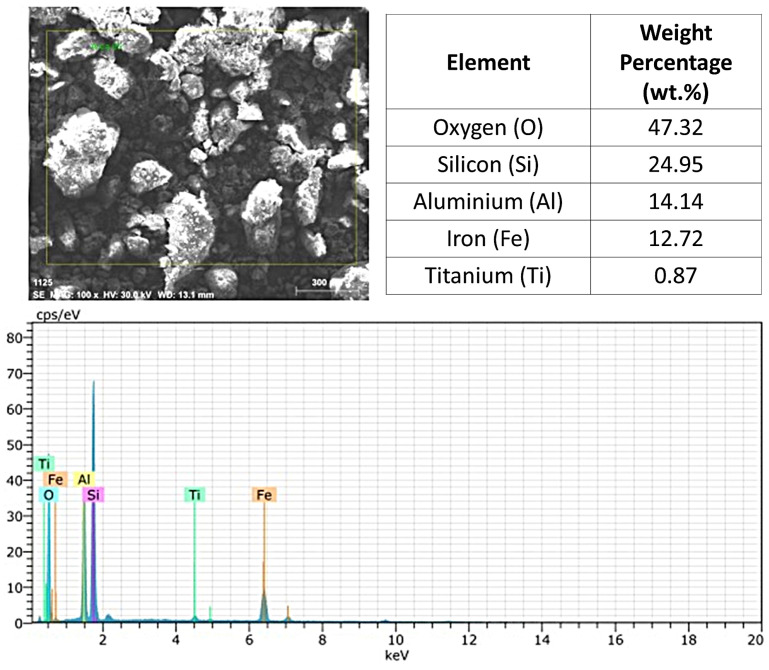
EDX analysis of laterite + sand.

The EDX analysis of the laterite + sand mixture indicated the presence of the following elements and their respective percentages: O (47.32%), Si (24.95%), Al (14.14%), Fe (12.72%), and Ti (0.87%), as illustrated in Figure 8. The proportion of silicon significantly increased, reflecting the addition of sand to the mixture. This alteration led to a decrease in the aluminum and iron concentrations, highlighting the dilution of sesquioxides, which are crucial for the cementation and mechanical strength of the material. The lower concentration of iron may result in less cementation compared to pure laterite, potentially adversely affecting its suitability for pavement applications.

The comparative analysis of the two samples shows that the Si/Al ratio is significantly higher in the mixture (1.76%) than in the pure laterite (1.14%), suggesting a potential change in physical and mechanical properties. The increase in silicon may lead to higher plasticity in the material, making it less suitable for applications requiring high stability, such as base layers in pavements. Additionally, the titanium content in the pure laterite was 1.50%, while in the laterite–sand mixture, it decreased to 0.87%. Although present in low concentrations, titanium can influence the strength and durability of the material.

The reduction in titanium content in the mixture may affect the material’s performance, particularly under prolonged exposure to severe environmental conditions. This raises questions regarding the suitability of the mixture for applications that demand weather resistance. The change in chemical composition caused by the addition of sand to laterite could result in a decrease in sesquioxide levels and an increase in silica levels. Considering the research conducted by Medina et al. [40], which highlights the importance of the chemical composition of lateritic soils concerning their mechanical and hydraulic behavior, substituting the chemical components that contribute to the soil’s good mechanical performance with sand may lead to significant losses. Thus, the interaction between the mixture’s components could substantially affect the material’s performance, especially in paving applications.

**Figure 9:**
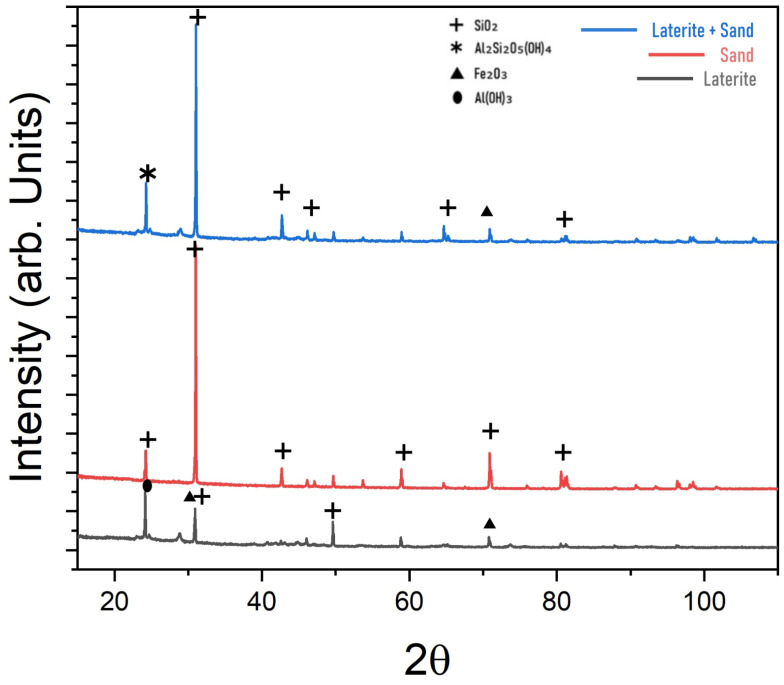
XRD analysis of pure laterite and laterite + sand.

The XRD analysis presented in Figure 9 shows that both the pure laterite and the laterite–sand mixture exhibit quartz (SiO_2_) as the predominant mineral, with distinct peaks around 26.6° (2θ). Additionally, kaolinite (Al_2_Si_2_O_5_(OH)_4_) and hematite (Fe_2_O_3_) were identified, confirming the presence of clays and iron oxides typical of lateritic soils. The addition of sand intensified the quartz peaks and may have reduced the relative intensity of the hematite peaks, suggesting a lower presence of iron oxides in the mixture. Thus, this modification in mineral composition may indicate a slight dilution of the lateritic characteristics, which could influence the mixture’s color, mechanical properties, and durability.

### 3.4. Mechanical Analysis

#### 3.4.1. Resilient Modulus

The regression parameters are presented in Table 3 with the values of the coefficient of determination ($R^{2}$) and the value of the linear resilient modulus for each sample, that is, the average of the RM values obtained at each stress pair after performing mathematical modeling.

The composite model was selected for this study due to its appropriate coefficient of determination and simplicity, as shown in Equation (1). Moreover, this model aligns with the Brazilian mechanistic–empirical pavement design method (MeDiNa), which is used for characterizing the stiffness of subgrade soils and granular materials [41,42,43,44,45].

In this model, the coefficient $k_{2}$, associated with confining stress, significantly influences the RM value more than $k_{3}$, which is linked to deviator stress. However, a positive value of $k_{3}$ suggests that increasing deviator stress increases the resilient modulus.

(1) $$RM=k_{1}\cdot \sigma_{3}^{k_{2}}\cdot \sigma_{d}^{k_{3}}$$

The variation in the MR ranges from 744 to 790 for the 18 applied stress pairs, achieving a good fit for the composite model with R² = 0.9956 and 0.9822, respectively. These values are suitable for use in pavement applications and are higher than those determined by Bernucci et al. [46] for a simple graded aggregate (100 to 400 MPa) and the range proposed by Balbo [47] (200 to 350 MPa). The MR values are also comparable to those of laterite gravel from the state of Acre, Brazil (566 to 585 MPa) [2], as well as those of laterite from the state of Pará, Brazil, reported by Veloso [13] (325 to 836 MPa).

Although the addition of sand is commonly used to adjust soil gradation and meet regulatory requirements, studies such as those by Madu [15], Okonkwo et al. [17], and Consoli et al. [14] suggest that it can improve properties like the CBR. However, in our MR results from the triaxial repeated load tests, no significant improvements were observed with sand addition. On the contrary, the natural laterite exhibited a slightly better performance, suggesting that the reduction in titanium content, which plays a key role in the formation of sesquioxides responsible for the cementation of lateritic soils, negatively impacted the material’s cementitious properties. This decrease in sesquioxides, particularly due to the reduction in titanium, may impair the material’s mechanical performance. Studies such as those by Medina et al. [40] emphasize the importance of the chemical composition of lateritic soils in relation to their physical properties, and how changes to this composition, especially reductions in cementing components like titanium, can significantly affect performance, particularly in paving applications.

#### 3.4.2. Permanent Deformation (PD)

**Figure 10:**
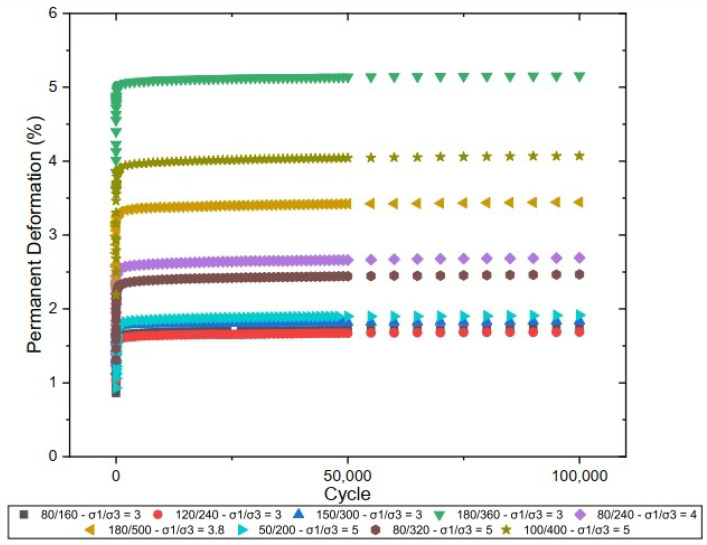
Permanent deformation of laterite + sand.

Figure 10 illustrates the behavior of the studied material concerning PD, comparing the results obtained for specimens of laterite with added sand.

It is observed that samples subjected to higher stress levels exhibit more pronounced deformation in the initial cycles, followed by a progressive stabilization as the number of cycles increases. This behavior suggests a structural accommodation of the particles after the initial cycles, with the accumulation of PD significantly decreasing after approximately 50,000 cycles. The variation in deformation accumulation clearly demonstrates the influence of the stress ratio, indicating that proper control of the applied stresses can optimize the performance of the layers in terms of resistance to PD.

**Figure 11:**
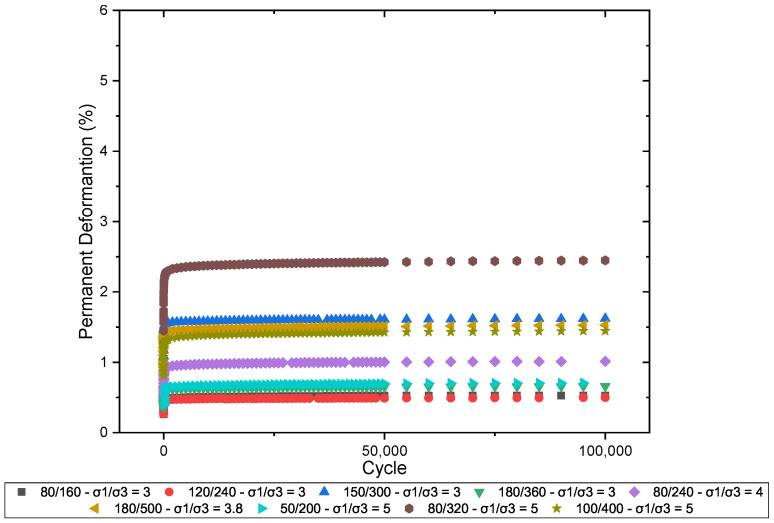
Permanent deformation of laterite.

Figure 11 illustrates the behavior of PD in pure laterite when subjected to different stress ratios ($\sigma_{1}$/$\sigma_{3}$). The pure laterite exhibited lower accumulated deformation values than the laterite + sand mixture. It is observed that the pure laterite stabilizes at lower levels of permanent deformation, with values below 2.5%, even under the highest applied stresses. In comparison to the behavior of the sand mixture, the isolated laterite demonstrated greater resistance to deformation during the initial cycles, likely due to its higher cohesion and internal strength. However, both the laterite and the sand mixture exhibit a structural accommodation behavior after the first 10,000 cycles, suggesting that incorporating sand into the gradation matrix influences the initial accumulation of deformation but does not significantly affect the long-term behavior.

Figure 10 and Figure 11 also indicate that the PD increases with the deviator stress. For instance, the stress ratio ($\sigma_{1}$/$\sigma_{3}$) = 5 caused a total plastic displacement more than 300% larger than the ($\sigma_{1}$/$\sigma_{3}$) = 3 at N = 100,000. This indicates a nonlinear relationship between the deviator stress and the resistance to permanent displacements and infers that the materials are more susceptible to plastic responses for larger deviator stresses. Other authors have already observed this type of behavior for fine lateritic materials (e.g., Guimarães et al. [48]; Lima et al. [3]; and Guimarães et al. [2]).

An interesting way to analyze the behavior of soils in relation to PD is to utilize the parameters from Guimarães’s model [49], which were reported for different stress states: low ($\sigma_{d}$ = 0.7 kgf/${\mathrm{cm}}^{2}$ and $\sigma_{3}$ = 0.7 kgf/${\mathrm{cm}}^{2}$), medium ($\sigma_{d}$ = 1.0 kgf/${\mathrm{cm}}^{2}$ and $\sigma_{3}$ = 3.0 kgf/${\mathrm{cm}}^{2}$), and high ($\sigma_{d}$ = 1.4 kgf/${\mathrm{cm}}^{2}$ and $\sigma_{3}$ = 4.5 kgf/${\mathrm{cm}}^{2}$).

**Figure 12:**
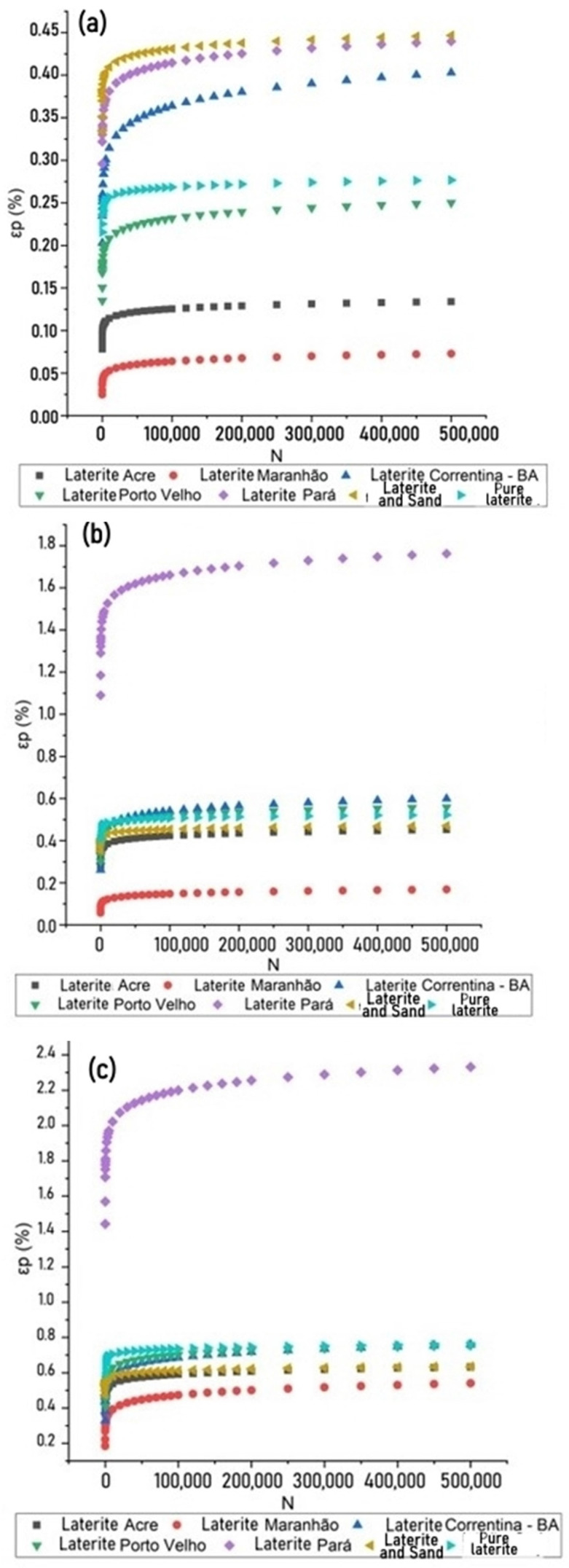
Permanent deformation of laterites.(**a**) low stress state; (**b**) medium stress state; (**c**) high stress state.

Figure 12 presents comparisons of the permanent deformation models for pure laterite and laterite + sand from this study with models of lateritic soils from Acre, Pará, Correntina (BA), and Maranhão and Porto Velho, as investigated by Guimarães et al. [2], Veloso et al. [13], and Guimarães, Santos, and Motta [50], respectively.

In general, pure laterite and the laterite–sand mixture demonstrated competitive performance compared to the other analyzed laterites. The pure laterite exhibited higher resistance under low-stress states, while the laterite + sand mixture showed superior behavior under medium and high stresses. However, under medium and high stresses, the pure laterite displayed behavior similar to that of the laterite–sand mixture, showing a significant capacity to withstand the applied loads, albeit with a slight inferiority compared to the mixture.

The laterite from Correntina, BA, demonstrated the best overall performance, with the lowest accumulation of permanent deformation across all stress states. In contrast, the laterites from Pará and Porto Velho exhibited the highest rates of permanent deformation, indicating lower resistance to repeated loading cycles. Both mixtures analyzed in this study showed similar behavior regarding stabilization after the initial loading cycles. The pure laterite proved capable of adequate resistance, even under higher-stress conditions, suggesting that while adding sand contributes to better performance in higher-stress states, the pure laterite provides considerable resistance across various loading scenarios.

**Figure 13:**
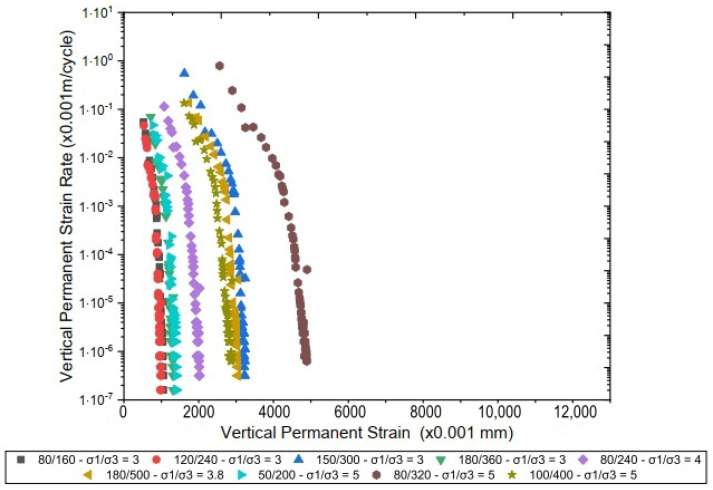
Permanent strain rate per accumulated strain of pure laterite.

**Figure 14:**
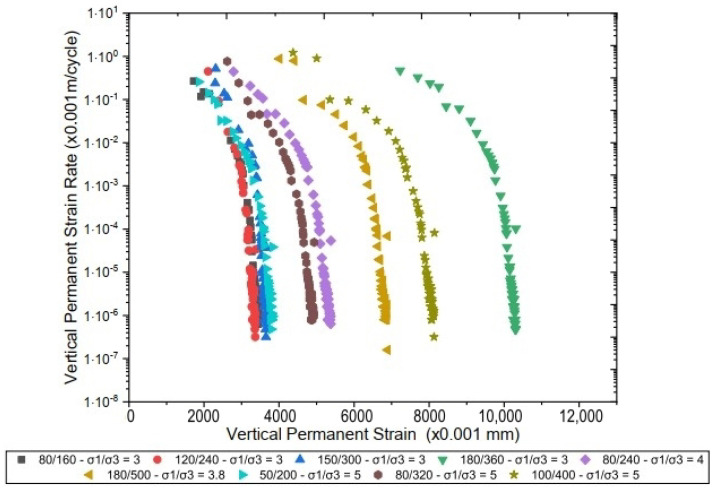
Permanent strain rate per accumulated strain of laterite + sand.

The “shakedown” analysis presented in Figure 13 and Figure 14 compares the permanent deformation behavior of pure laterite and the laterite–sand mixture, respectively. The results indicate fairly similar behaviors between the two materials despite subtle differences in their deformation accommodation behaviors.

In Figure 13, which illustrates the behavior of the pure laterite, we observe that four stress pairs (80/160, 120/240, 180/360, and 50/200 (σd/σ3, KPa/KPa)) achieve a permanent deformation rate of approximately $10^{-7}$ m/cycle, indicating a type A behavior for most loading scenarios, as classified by Werkmeister [33]. This type A behavior suggests that the material’s deformation tends to stabilize after an initial number of loading cycles, which is desirable for pavement layers.

However, under higher loading conditions, such as with the stress pair 180/500 (σd/σ3, KPa/KPa), the material exhibits a type AB behavior, with a slight progression of deformation. This indicates that, although the material does not achieve perfect stability under higher stresses, it still demonstrates acceptable performance with gradual accommodation over time.

In Figure 14, which presents the behavior of the laterite–sand mixture, the curves show behavior very similar to that of the pure laterite, with most stress conditions stabilizing over time. While type A behavior predominates, especially under lower stresses, type AB behavior is also observed under certain loading conditions, reinforcing the similarity between the two materials. It is important to note that adding sand to laterite generally aims to ensure that the material meets specific grain size requirements outlined in various regulatory criteria, remains within Atterberg limits, and adheres to conventional classification standards such as the USCS and TRB criteria. However, the behavior of pure laterite demonstrates sufficient stability for use in pavement layers. The accommodation trend observed in both materials, even without additional stabilization, suggests that pure laterite could be successfully employed in pavement construction, as its accommodation behavior is suitable for withstanding repeated loads while minimizing excessive deformations.

Furthermore, based on the analysis of permanent deformation and resilient modulus tests, both pure laterite and the laterite–sand mixture can be classified as type IVa or IVb laterites (i.e., exhibiting excellent performance, with a high RM and low PD or with an RM lower than 300 MPa and a high PD, respectively) according to the classification proposed by Guimarães et al. [51]. This category encompasses laterites that, while not fully meeting the grain size criteria outlined in the DNIT 098 standard [22], exhibit superior mechanical behavior characterized by a high resilient modulus (MR > 700 MPa) and a tendency for accommodation in PD.

## 4. Conclusions

This study evaluated the mechanical characteristics of a laterite from the state of Roraima, an area rich in this soil type. Specifically, it aimed to investigate the effect of sand addition on the material’s mechanical properties, providing a more in-depth analysis to overcome the limitations of conventional classification and usage approaches in pavement applications.

The main conclusions obtained by this research are listed as follows:

- Analyses from the perspective of pavement mechanics revealed that adding sand does not necessarily enhance mechanical performance. This is evidenced by the resilience modulus (MR) values, where the laterite + sand mixture exhibited an average MR of 744 MPa, while the pure laterite achieved an average MR of 790 MPa. These data suggest that while adding sand may improve grain size characteristics, it does not significantly increase stiffness, highlighting the need to refine this practice to ensure its effectiveness in pavement applications.
- The results of the permanent deformation tests indicated that the laterite–sand mixture exhibited approximately twice the plastic displacement of pure laterite under similar loading conditions. For instance, under stresses of 180/500 (σd/σ3, kPa/kPa), pure laterite demonstrated a lower progression of deformation and a higher capacity to withstand repeated loads without excessive deformations. These findings reinforce the ability of pure laterite to meet the mechanical requirements for pavements without the need for additional granulometric adjustments.
- The shakedown analysis revealed that pure laterite predominantly exhibited type A behavior (deformation stabilization) for most of the stress pairs evaluated, achieving permanent strain rates of up to $10^{-7}$ m/cycle under conditions such as 80/160, 120/240, and 50/200 (σd/σ3, kPa/kPa). At higher stress levels, a type AB behavior was observed, indicating progressive accommodation, but still remaining within acceptable limits. The laterite–sand mixture exhibited similar behavior but with higher permanent strain rates, once again highlighting the greater stability of pure laterite.

The results of this study highlight the potential of pure laterite for use in pavement layers, demonstrating that, under certain conditions, the material can be employed without the need for granulometric adjustments or the addition of other components. Mechanical analysis, such as triaxial testing, is essential to evaluate soil behavior and ensure adequate pavement performance. The data reported here suggest that laterite, in its natural state, can provide the necessary properties for satisfactory structural performance without the intervention of additional materials.

## Figures and Tables

**Table 1:** Test parameters adapted from BS EN 13286-7 [31].

Test	$\sigma_{3}$ (kPa)	$\sigma_{d}$ (kPa)	$\sigma_{1}/\sigma_{3}$
1	80	160	3
2	120	240	3
3	150	300	3
4	180	360	3
5	80	240	4
6	180	500 *	3.78
7	50	200	5
8	80	320	5
9	100	400	5
10 *	30	150	6
11 *	70	250	6
12 *	100	500	6

* Indicates tests not carried out, according to the limitations of the scope of the study.

**Table 2:** Geotechnical characterization.

Sample	LL (%)	PL (%)	PI (%)	SUCS	TBR	$\rho_{\mathrm{dmax}}$ $\left({g/\mathrm{cm}}^{3}\right)$	Wot (%)
Laterite soil	44	21	23	GC	A-2-7	1.99	13.1
Laterite soil + Sand	23	13	10	SC	A-2-4	2.16	9.86

**Table 3:** Parameters of average MR using the composite model.

Material	RM Medium (MPa)	k1	k2	k3	R^2^
Laterite + Sand	744	160.67	2177.63	0.9231	0.9956
Laterite	790	378.59	1781.47	0.5994	0.9822

## Data Availability

The original contributions presented in this study are included in the article; further inquiries can be directed to the corresponding authors.
